# A detailed investigation of dielectric-modulated dual-gate TMD FET based label-free biosensor via analytical modelling

**DOI:** 10.1038/s41598-022-24677-6

**Published:** 2022-12-07

**Authors:** Monika Kumari, Niraj Kumar Singh, Manodipan Sahoo

**Affiliations:** Department of Electronics Engineering, Indian Institute of Technology (Indian School of Mines), Dhanbad, 826004 India

**Keywords:** Nanoscale devices, Nanoscale materials, Electrical and electronic engineering, Materials for devices

## Abstract

In this work, an analytical model is developed for DM-DG-TMD-FET- based Biosensor including Fringing-field effects. The Analytical model has been developed for two different Device structures, namely Device structure-1 (without a gate above the nano-cavity) and Device structure-2 (with a gate above the nano-cavity) based on modulation of the dielectric constant of biomolecules in the nano-cavity region. The proposed model has been validated against both numerical quantum simulation results with the help of a few fitting parameters and it also agrees with the 2-dimensional numeric simulator SILVACO TCAD used in this work. The presence/absence of biomolecules has been detected by the metric of threshold voltage sensitivity $S_{Vth}$ and drain current $I_{d}$ for the neutral as well as charged biomolecules. Sensitivities of partially filled nano-cavities arising out of steric hindrance in both the biosensors are compared. Optimization of device dimensions has also been included in this work to enhance the sensitivity of the biosensors. It has been witnessed that the sensitivity of the proposed biosensor is $\sim$ 100% higher in Device structure-1 for neutral biomolecules with dielectric constant $\kappa$ = 12, when compared to Device structure-2 for fully filled cavities. Whereas for the charged biomolecules, Device structure-1 shows $\sim$ 50% enhanced sensitivity than Device structure-2 for $N_{f}=-1\times 10^{-12}$ $\text{C}/\text{cm}^2$. Device structure-1 demonstrates $\sim$120% higher sensitivity than Device structure-2 with partially filled cavities (i.e. 66% filled cavity). Finally, benchmarking of the proposed biosensor is presented with contemporary, state-of-the-art biosensors and it is highlighted that $MoS_{2}$ FET-based biosensor emerges with a superior sensitivity of $S_{Vth}$ = 0.81 V for $\kappa = 12$.

## Introduction

FET-based biosensors have been extensively investigated to detect a range of target biomolecules due to their superior sensitivity, swift label-free detection capabilities, low power consumption, compatibility with CMOS technology, and large scale production at a low cost. These factors have become more critical as the demand for ultrasensitive biosensors has risen in fields such as medicine, agriculture, defence, and environmental monitoring, among others^1–3^. Among FET-based biosensors, Dielectric Modulated (DM) FETs have shown better sensitivity and are mostly preferred in the field of biosensing applications as the created nano-cavities provide sufficient volume and efficient conjugation of biomolecules which results in enhancement in sensitivity^4^. Choi et al. demonstrated the first DM-FET-based biosensor with nano-cavities for label-free detection of biomolecules^2^. After that many dielectric-modulated FET-based biosensors have been reported with the enhancement in sensitivity^5–9^.

FET-based biosensors with 1-D Nanowires and CNTs as channel material have a significant advantage over label-based detection^10^ because they exhibit good electrostatics between biomolecules and the channel, resulting in increased sensitivity. However, synthesizing biosensors that meet all of these criteria at the same time has proven difficult, as 0-D and 1-D nanomaterials-based biosensors are difficult to fabricate on a large scale and have high production costs^11–13^, limiting the practical feasibility of such structures.

Contrarily, tremendous progress has recently been made with Transition Metal Dichalcogenides (TMDs), one of the most intriguing 2-D semiconducting materials for next-generation biosensors. Because of their excellent electrostatic control, planar nature, high surface-to-volume ratio, superior charge sensitivity, and high electron mobility, among other advantage, TMDs hold a lot of promise for use as a channel in FET-based ultra-sensitive biosensors^12,14^ .

Furthermore, due to their comparably weak interlayer connections, 2-D materials have a lower surface roughness than 3-D materials^15^. TMD materials unlike Graphene exhibit a band-gap that is essential for the operation of FET-based biosensors since the binding process at the interface between the channel and biomolecules modulates the carrier transport in 2-D layered materials^16^.

Moreover, in recent years the application of various 2-D layered materials like $MoS_{2}$, $WSe_{2}$ and $MoSe_{2}$ in FET-based biosensors has become extremely promising. $MoSe_{2}$ has smaller band-gap than $MoS_{2}$ and $WSe_{2}$ but it exhibits larger reduced effective mass which is preferable for Tunnel FET-based applications^17,18^. Whereas, $WSe_{2}$-based biosensors exhibit higher linear-regime sensitivities in comparison with $MoS_{2}$-based biosensors^19^. Because of the recent advances in the synthesis of $MoS_{2}$ sheets using CVD and liquid phase exfoliation techniques^12,16^, as well as their excellent compatibility with commercial planar processes for large scale production^15,20^, $MoS_{2}$ FET-based biosensors are widely investigated among TMD materials.

Sarkar et al. have reported detection of streptadavin using $MoS_{2}$ FET-based biosensor with $HfO_{2}$ as gate dielectric functionalized with biotin^11^. Wang et al. have reported APTES functionalized $MoS_{2}$ nanosheet-based biosensor for the cancer marker protein detection^21^. Nam et al. have reported $MoS_{2}$ FET-based biosensor functionalized with $TNF {\text{-}}\alpha$ anti-bodies for sensing of $TNF{\text{-}}\alpha$ molecules^22^.

However, in order to further investigate 2-D material based FET in biosensing applications, an analytical I-V model that accounts for realistic circumstances when conjugating the biomolecules in the nano-cavities, which seem to be unavoidable during fabrication, is required. Unfortunately, little progress has been made in developing various analytical models for DM-TMD-FET-based biosensors. An I-V model has been developed by Rahman et al.^23^ for single-sided cavity, $MoS{_2}$-based biosensor. However, this model does not take into account fringing-field effects, which in sub-100 nm FETs cannot be disregarded. Furthermore, the author has investigated the effect on sensitivities when the nano-cavities are filled completely, which is practically impossible.

Main contributions of this work can be summarized as: an analytical model of DM-TMD-FET-based biosensor has been developed including several design parameters like nano-cavity thickness, dielectric constant and charge of biomolecules. The veracity of the model has been verified with experimental data^24^ and 2-D numeric simulator SILVACO TCAD^25^. Two device structures namely, Device structure-1 (without gate above the nano-cavity) and Device structure-2 (with gate above the nano-cavity) have been investigated incorporating fringing-field effects in the model and its effect on the characteristics of the biosensors has been analyzed. Sensitivity metrics such as threshold voltage $V_{th}$, shift in threshold voltage $\Delta$ $V_{th}$ and drain current are estimated for neutral biomolecules as well as charged biomolecules. Partially filled nano-cavities with different filling factors have also been considered in this work and the analytical model has been developed to incorporate this. The effect of steric hindrance including concave, convex, decreasing, and increasing step profiles in the nano-cavity has been considered and the impact of these profiles on the sensitivity has been observed.

## Device structure

**Figure 1:**
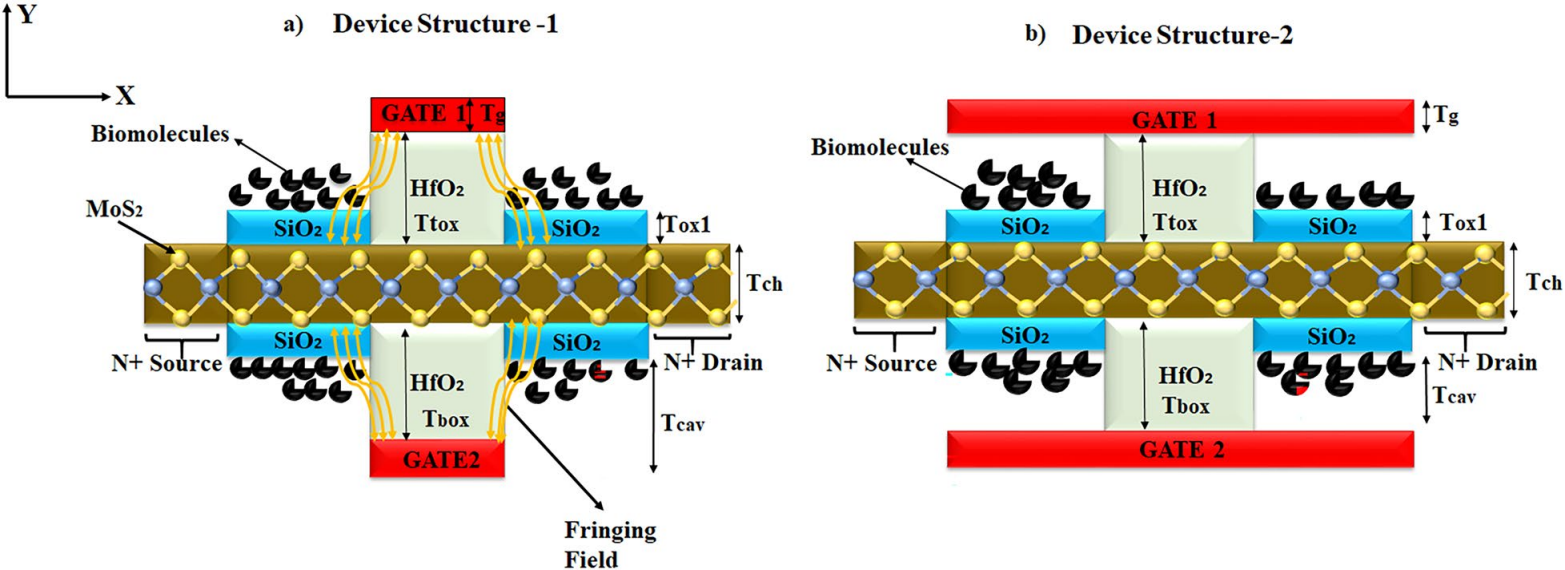
(**a**) Device structure-1 of dielectric-modulated, dual-gate, TMD FET (DM-DG-TMD-FET)-based Biosensor, and (**b**) Device structure-2 of DM-DG-TMD-FET-based biosensor.

Figure 1a,b show the schematic of the proposed monolayer TMD-FET-based biosensor without gate over cavity and with gate over cavity.

**Figure 2:**
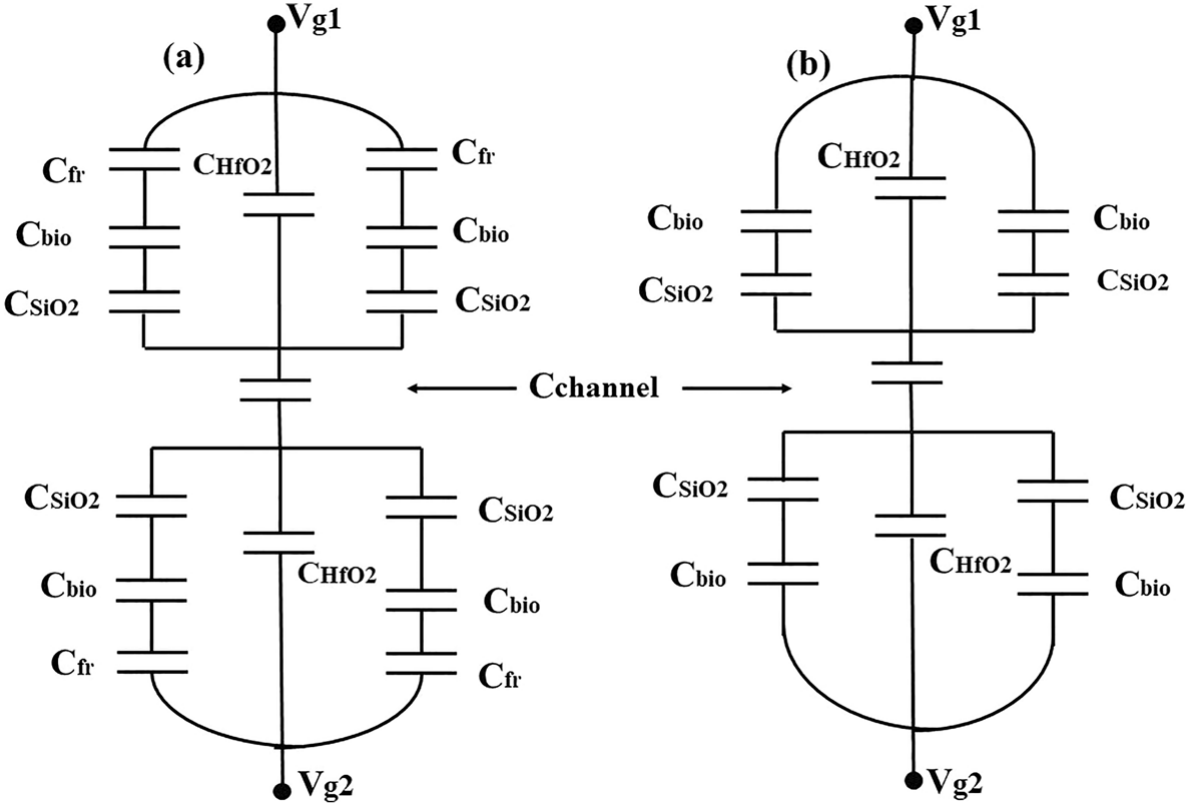
Equivalent Capacitance Model for (**a**) Device structure-1, (**b**) Device structure-2.

**Figure 3:**
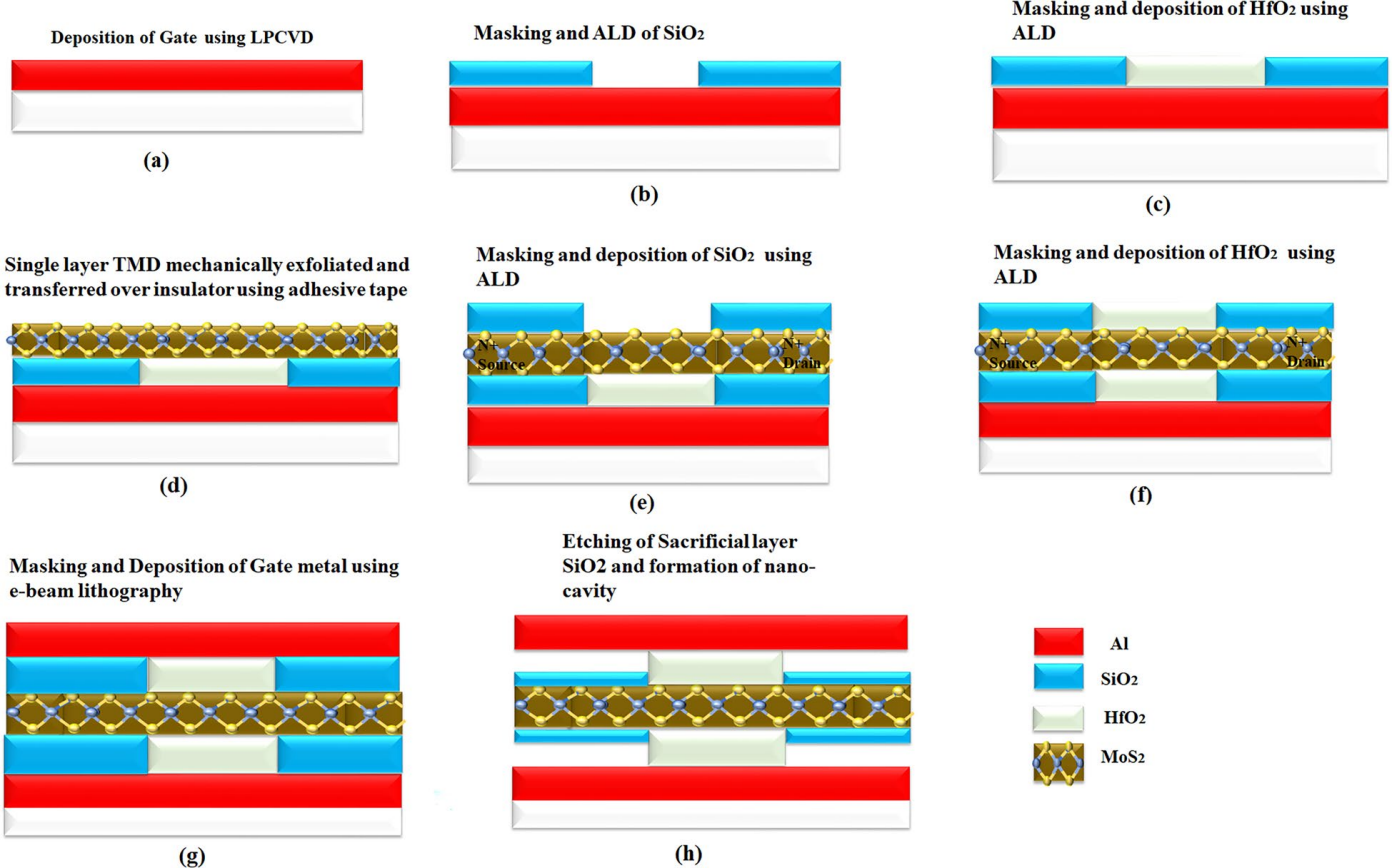
Proposed fabrication process of the DM-DG-TMD-FET-based biosensor.

An equivalent capacitance model of the device structure-1 and 2 has also been drawn in Fig. 2. Here, *x* is the channel direction and, *y* is perpendicular to the channel. The channel region is divided into three parts, regions at both the source ($L_{1}$) and the drain side ($L_{3}$) work as cavity, where biomolecules can be immobilized, region beneath the high-$\kappa$ gate oxide is the overlap region, ($L_{2}$). A $SiO_{2}$ layer having thickness 1 nm is taken in the cavity regions, as illustrated in Fig. 1, which acts as an adhesion layer for the biomolecules^3^. Since, $MoS_{2}$-based biosensor has been widely explored^26,27^ in the recent years, it has been utilized as a channel material for performing various analysis in this work. Other TMD materials like, $WSe_{2}$ and $MoSe_{2}$ can also be deployed as channel materials^28^. TMD-FET-based biosensor under consideration consists of a monolayer p-doped $MoS_{2}$ channel sandwiched between the top and the bottom $HfO_{2}$ layer. *Al* is used as top and bottom gates. Highly n-doped monolayer $MoS_{2}$ has been used in the source and the drain regions. The channel has a thickness and length of 0.7 nm^19^ and 60 nm respectively. On the source and drain sides, the cavity is 20 nm long, respectively. The top and bottom oxide layers are each 10 nm thick. The remaining model parameters are listed in Table 1. A tentative fabrication process^20,27,29^ of the proposed TMD FET-based biosensor has been depicted in Fig. 3.

**Table 1:** Device parameters of the proposed structure.

Symbol	Parameters	Range
$L_{ch}$	Length of the channel	100 nm
$L_{1},L_{3}$	Length of the nano-cavity	30 nm
$L_{g}$	Length of gates for device structure	60 nm
$T_{tox}$	Thickness of the front gate oxide	10 nm
$T_{box}$	Thickness of the back gate oxide	10 nm to 200 nm
$T_{cav}$	Thickness of the nano-cavity	10 nm
*W*.*F*	Work function of gates	4.1 eV
$N_{ch}$	Channel doping	$10^{18}$ $\text{cm}^{-2}$
$N_{sd}$	Source and drain doing	$10^{20}$ $\text{cm}^{-2}$

## Results and discussions

The characteristics of the DM-DG-TMD-FET-based biosensor for neutral and charged biomolecules are discussed in this section. The sensitivity of the biosensor is measured in terms of threshold voltage ($V_{th}$), variation in threshold voltage ($\Delta V_{th}$) and drain current ($I_{ds}$).

**Figure 4:**
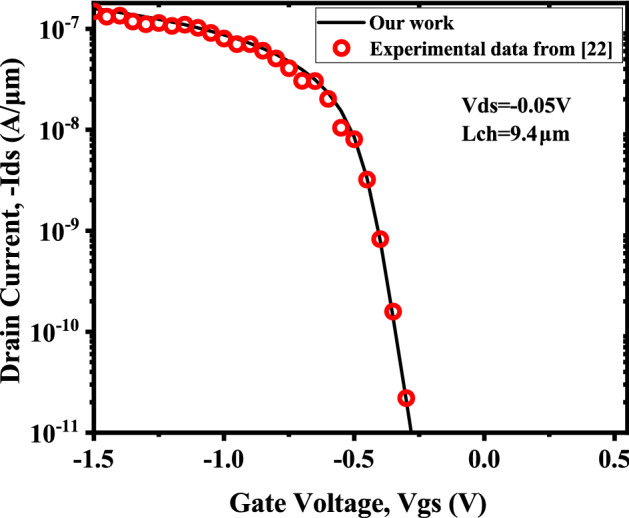
Drain current, $(I_{ds})$ versus Gate to source bias, $(V_{gs})$) for back-gate, (Vgb) − 40 V and drain to source bias, $V_{ds}$= − 0.05 V with channel-length, $L_{ch}$ = 9.4 nm, front and back gate oxides are $ZrO_{2}$ $T_{tox}=17.5\; \text{nm}$, $k_{tox}=12.5$ and $SiO_{2}$ $(T_{box}=270 \; \text{nm}, k_{box}= 3.9)$ respectively.

**Figure 5:**
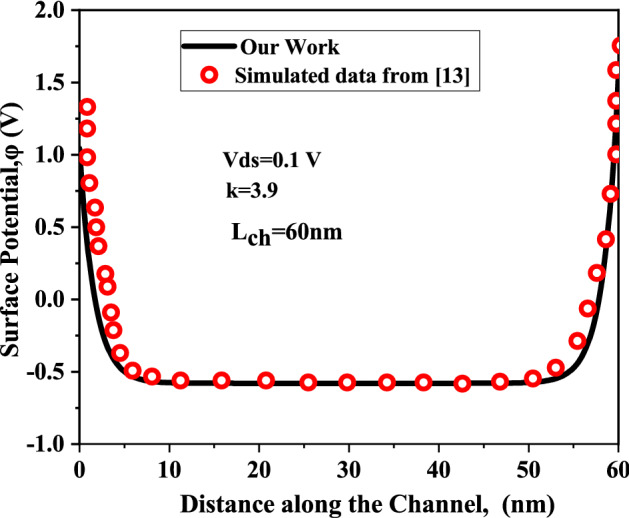
Surface potential calculated using our proposed model vs. quantum simulated data of^23^ for $V_{gs}=1 \; \text{V}$.

The proposed model is validated with the experimental I-V characteristics of monolayer $WSe_{2}$ by Fang et al.^24^, quantum simulation results obtained by Rahman et. al.^23^ and with two dimensional numerical simulator Silvaco TCAD^25^. Exactly same sets of the device and material parameters are utilized to validate our proposed model with the simulator. Figure 4 depicts the I-V characteristics of the DM-DG-TMD- FET- based biosensor obtained from the model along with the experimental results reported by Fang et al.^24^. Figure 5 shows the surface potential of DM-DG-TMD FET-based biosensor, which has been validated with self consistent NEGF simulation results of^23^. Drain current and surface potential shows a close match with the results from^24^ and^23^. Little discrepancy exists as a result of the various environmental set-up.

### Impact of neutral biomolecules and charged biomolecules on sensitivity

**Figure 6:**
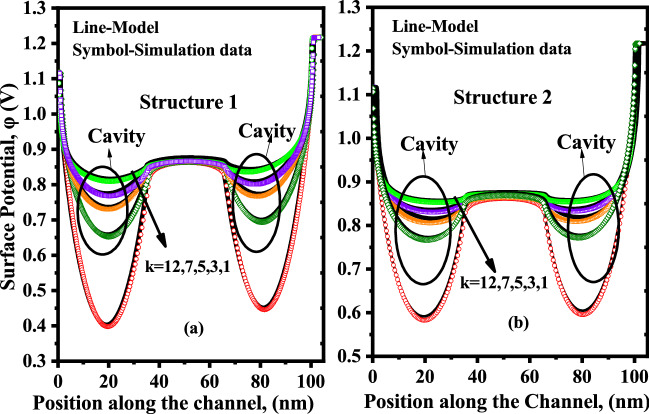
Surface potential of the proposed DM-DG-TMD-FET-based biosensor for (**a**) Device structure-1 and, (**b**) Device structure-2 with different dielectric constants ($\kappa =1,3,5,7,12$) of biomolecules. ($V_{gs}=0.1 \; \text{V}$,$V_{ds}=0.1 \; \text{V}$ $N_{f}=0$).

In this section, we have explored the impact of neutral biomolecules and charged biomolecules on the surface potential, threshold voltage $V_{th}$ and drain current of the proposed two structures of DM-DG-TMD-FET-based biosensor. Figure 6a shows the bending in surface potential when neutral biomolecules get immobilized in the cavity for the proposed DM-DG-TMD- FET-based biosensor, structure-1. In Device structure-1, there is no gate over nano-cavity so the effect of flat band voltage $V_{fb}$ is negligible and fringing capacitance plays a dominant role by influencing the surface potential. Deviation in the surface potential profile is clearly observed under the nano-cavity region, whereas, no deformation of the potential profile is observed in the region without a nano-cavity.

When the cavity is empty i.e filled with air ($\kappa =1$) the gate capacitance is low and the flat band voltage $V_{fb}$ in the cavity part is negligible so the voltage requirement will be more for the electrons to cross the source-channel barrier which is evident from (1). The $V_{th}$ of a MOSFET can be expressed as^30^

1 $$\begin{aligned} V_{th}= V_{fb}+2\varphi _{b}+\frac{q (\pm N_{f})}{C_{eff}} \end{aligned}$$

**Figure 7:**
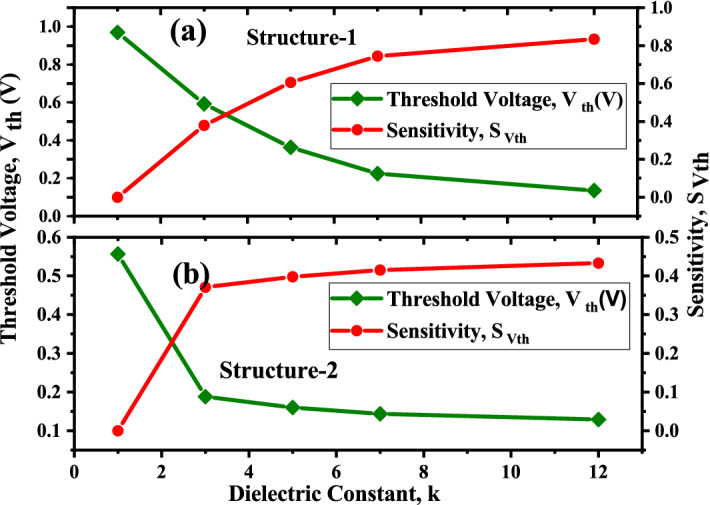
Threshold voltage and Sensitivity w.r.t to dielectric constant ($\kappa$) of biomolecules with for Device structure-1 and Device structure-2 ($V_{gs}=1.2 \; \text{V}$, $\kappa =5$, $N_{f}=0$).

Moreover, when the dielectric constant of the biomolecules increases, the effective gate capacitance rises along with fringing-field capacitance, changing the surface potential in the cavity region. Thus with an increase in the dielectric constant of biomolecules the fringing-field effects dominates and the surface potential barrier is reduced.

For the proposed Device structure-2, Fig. 6b illustrates the deformation in surface potential when neutral biomolecules get immobilised in the cavity. Since there is gate over cavity, fringing capacitance is not dominant in this case and the control of gate over channel is adequate, as the channel is p-type, there will be negative flat band voltage across the channel and the amount of threshold voltage for the electrons to cross the source channel barrier will be less. In this case also the surface potential barrier decreases with the increase in dielectric constants of the biomolecules but the reduction in barrier is more when compared to Device structure-1 because of the presence of gate control throughout the channel region and sufficient amount of flat band voltage $V_{fb}$.

When the target biomolecules are immobilized in the nano-cavities, device characteristics are modulated according to the quantity of target biomolecules. The threshold voltage $V_{th}$ and change in threshold voltage $S_{Vth}$ are the most commonly utilised sensing metrics for evaluating the effectiveness of the biosensors among the different parameters that are affected by the target biomolecules.

It is evident from Fig. 7a,b, that the threshold voltage requirement for Device structure-1 is higher than that for Device structure-2, as the gate control over the channel in structure-1 is less so the $V_{fb}$ is negligible in the cavity area and threshold voltage increases and due to the fringing effect there is more increase in threshold voltage. For Device structure-2 there is full gate control over the channel thus threshold voltage requirement is less. It is also evident from Fig. 7a,b, that the threshold voltage decreases with the increasing $\kappa$. When the cavity is empty (i.e $\kappa =1$), the threshold voltage achieved is high, i.e 0.969 *V* for structure-1 and and 0.569 *V* for structure-2.

Figure 7a,b plots the sensitivity of the proposed two structures of DM-DG-TMD-FET-based biosensors. The sensitivity of the biosensor is measured by a shift in threshold voltage ($S_{Vth}$) before and after immobilization of the biomolecules in the nano-cavity region. Thereby, it can be defined as^31^

2 $$\begin{aligned} S_{Vth}=\frac{V_{th}(\kappa =1)-V_{th}(\kappa >1)}{V_{th}(\kappa =1)} \end{aligned}$$

It can be observed that sensitivity of device structure-1 is $\sim 100\%$ more than the sensitivity of device structure-2. As in the proposed biosensor structure-1, fringing capacitance dominates the total capacitance which leads to increase the threshold voltage as well as overall enhancement in the sensitivity.

**Figure 8:**
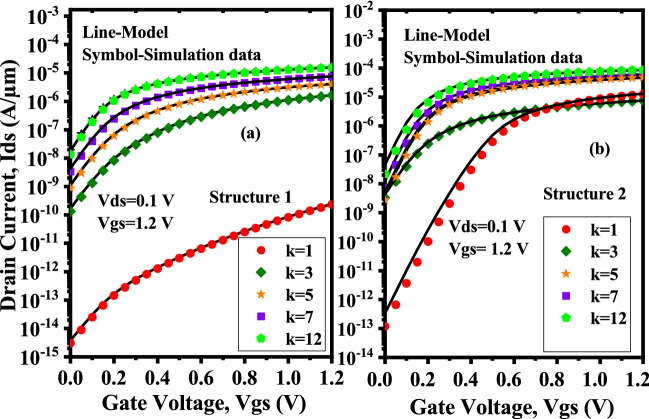
Variation in drain current w.r.t to gate bias for (**a**) Device structure-1 and (**b**) Device structure-2 ($V_{gs}=1.2 \; \text{V}$, $V_{ds}=0.1 \; \text{V}$, dielectric constants, $\kappa =1,3,5,7,12$, $N_{f}=0$).

Figure 8a shows the drain current variation when neutral biomolecules are conjugated in the nano-cavity region of the proposed Device structure-1. The OFF current of the device is minimum when the cavity is empty and it increases when the dielectric constant $\kappa$ in the cavity region increases, whereas, there is slightly increment in ON current. Now, comparing Device structure-2 Fig. 8b to Device structure-1, the ON and OFF current increases as the dielectric constants $\kappa$ increase. The variation in OFF current is greater than the variation in ON current because FETs conduct extremely low current in the subthreshold regime, so a smaller change in potential due to biomolecule immobilization leads to a reasonable change in subthreshold current, which is preferable for sensing operation.

**Figure 9:**
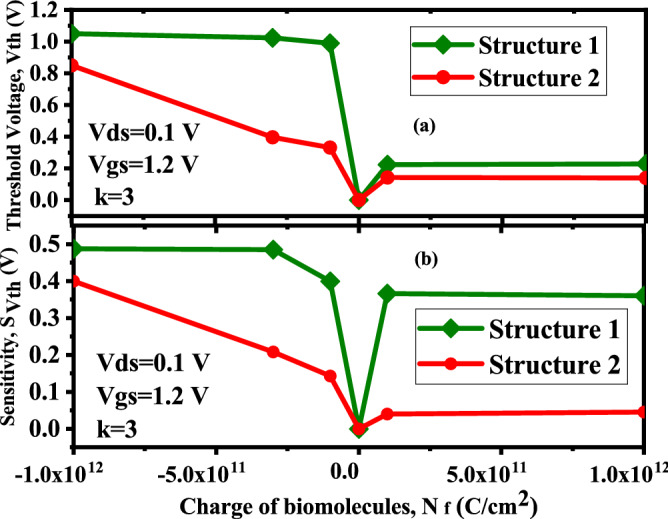
Threshold voltage and Sensitivity w.r.t to negatively and positively charged biomolecules for Device structure-1 and Device structure-2 ($V_{gs}=1.2 \; \text{V}$, $V_{ds}=0.1 \; \text{V}$ for $\kappa =3$).

Figure 9a,b shows the effect of charged biomolecules on the threshold voltage of the proposed structure. The impact of charged biomolecules on the sensitivity of the device is also depicted in Fig. 9a and b for both the proposed structures. The sensitivity parameter $S_{Vth}$ for negatively charged biomolecules in the figure is considerably higher than that for positively charged biomolecules. Additionally, the sensing metric rises in accordance with an increase in the charge density of negatively charged biomolecules. Sensitivity, on the other hand, first show a linear trend with an increase in charge density after that it get saturated to further variations. Before and after immobilising charged molecules, the proposed device’s sensitivity can be described as,

3 $$\begin{aligned} SV_{th}= \vert \frac{V_{th}(Neutral-biomolecules )-V_{th}(Charged-biomolecules)}{V_{th}(Neutral- biomolecules)}\vert \end{aligned}$$

**Figure 10:**
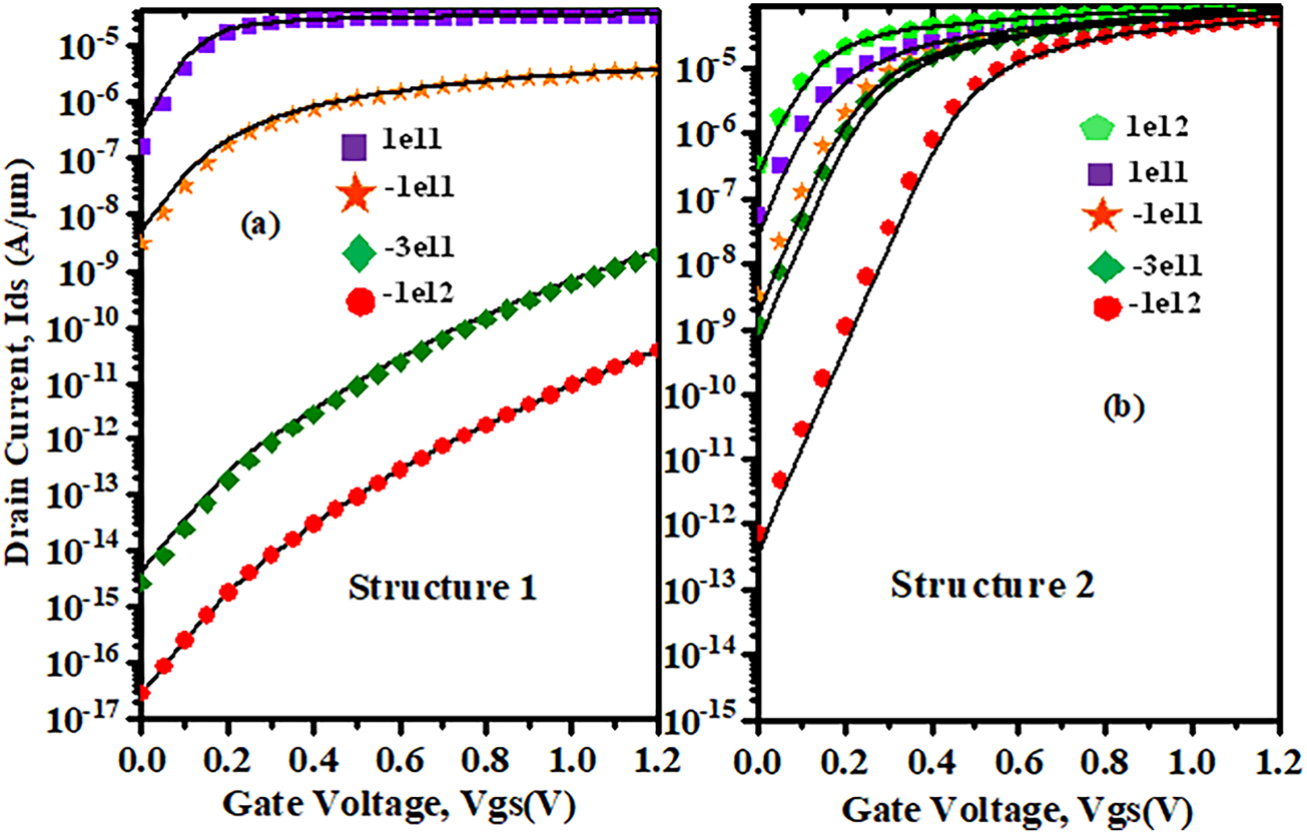
Variation in drain current w.r.t to gate bias for (**a**) Device structure-1 and (**b**) Device Structure-2 ($V_{gs}=1.2 \; \text{V}$ $V_{ds}=0\; \text{V}$ for negatively and positively charged biomolecules for $\kappa =3$).

Since, there is gate over the nano-cavity (Device structure-2) regions, thus, the threshold voltage requirement to deplete the channel will reduce by the amount of $V_{fb}$ compared to Device structure-1 for negatively/positively charged biomolecules, which can be understood from (20). For negatively charged biomolecules the threshold voltage requirement to deplete the channel is more and it is even more for device structure-1 because of the presence of fringing field effects. Which leads to decrement in ON and OFF current for negatively charged biomolecules. Whereas, for positively charged biomolecules, the threshold voltage requirement to deplete the channel is less for both device structures and it is even lesser for Device structure-2 because of the absence of fringing effects. Which leads to increment in ON and OFF current as shown in Fig. 10.

### Sensitivity analysis with the variation in thickness and length of the nanogap cavity

**Figure 11:**
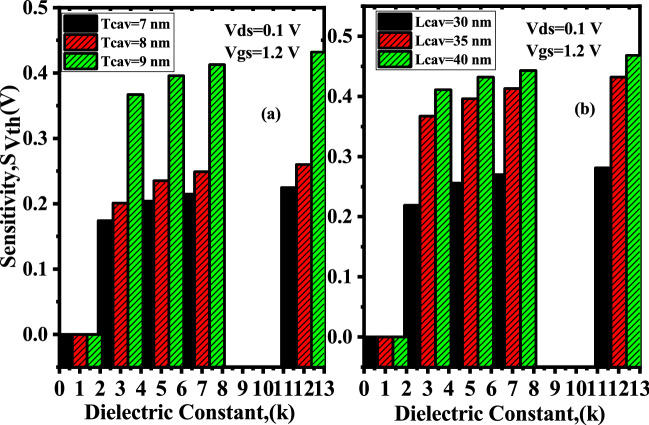
Sensitivity of the device for (**a**) Variation in cavity thickness (**b**) Variation cavity length ($V_{gs}=1,2 \; \text{V}$, $V_{ds}=0.1 \; \text{V}$, $\kappa =5$, $N_{f}=1\times 10^{11}$ $\text{C}/\text{cm}^2$).

In this section we have demonstrated how the device’s sensitivity varies with the thickness $T_{cav}$ and length $L_{cav}$ of the cavity. Figure 11a shows the change in threshold voltage $S_{Vth}$ with the variation in thickness of the cavity from 7 to 9 nm. With the increase in thickness of the cavity, barrier between source/channel junction increases, results in decrement of the drain current so, as expected $V_{th}$ linearly increases as $T_{cav}$ becomes thicker. The variation in sensitivity parameter with cavity length $L_{cav}$ varying from 30 to 40 nm of channel is shown in Fig. 11b. As the cavity length $L_{cav}$ increases, requirement of threshold voltage increases which leads to low drain current and higher sensitivity, which further improves if the channel length is large as also reported for DM-FET^6^.

### Impact of partially filled biomolecules on sensitivity

All of the preceding sections assumed that the cavities were completely filled with biomolecules, but in reality, entirely filled cavities are rarely possible.

**Figure 12:**
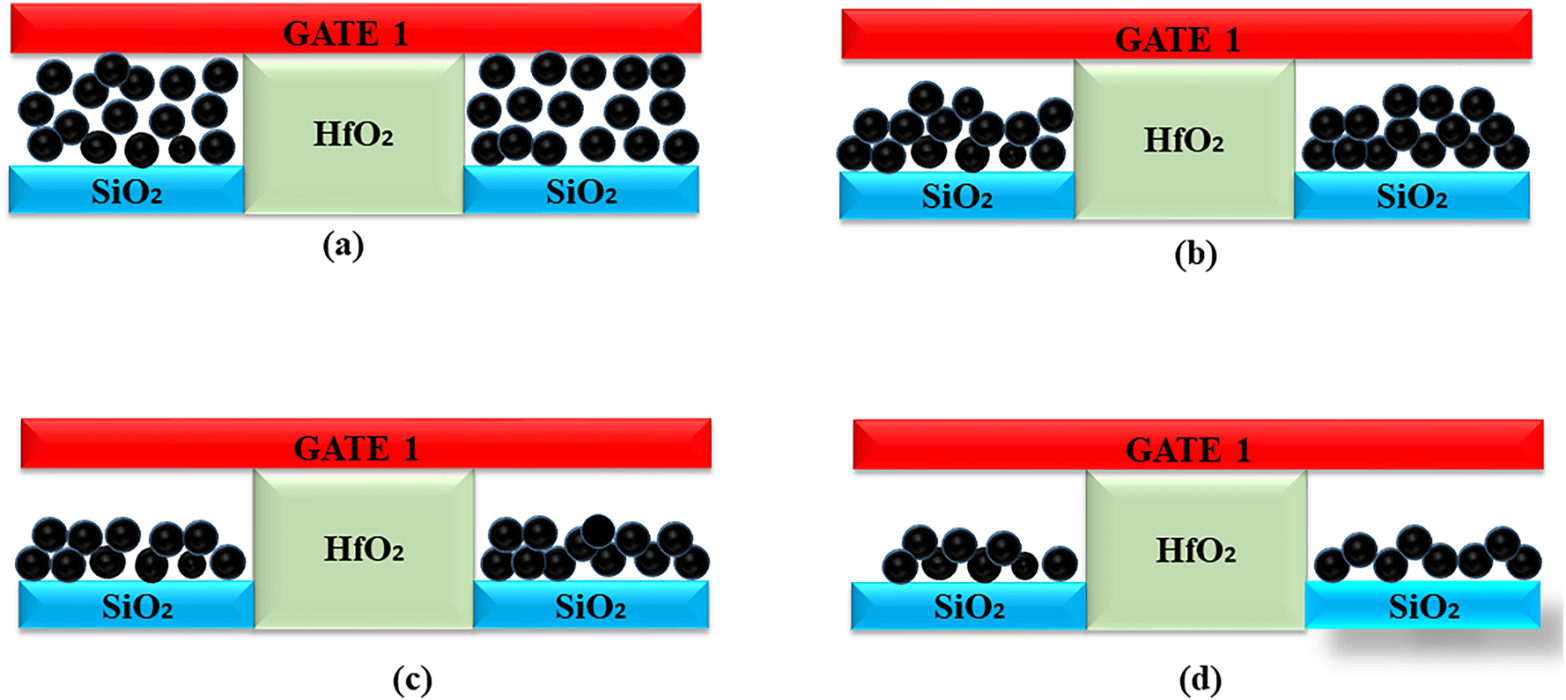
(**a**) Completely filled cavity (**b**) 66% filled cavity (**c**) 50% filled cavity (**d**) 33% filled cavity.

Thus, three distinct assumptions of biomolecules filling the nano-cavity are investigated here, as illustrated in Fig. 12. The fill-factor of the nano-cavity is defined as the ratio of the area covered by conjugated biomolecules to the overall area of the nano-cavity in percentage. This section discusses the effect of percentage volume filling of the nano-cavity on the sensitivity of DM-DG-TMD-FET-based biosensor. The fill-factor ($\rho _{T_{bio}}$) defined here as,

4 $$\begin{aligned} \rho _{T_{bio}}=\frac{T_{bio}(partially-filled)}{T_{bio}(fully-filled)} \times 100 \end{aligned}$$

**Figure 13:**
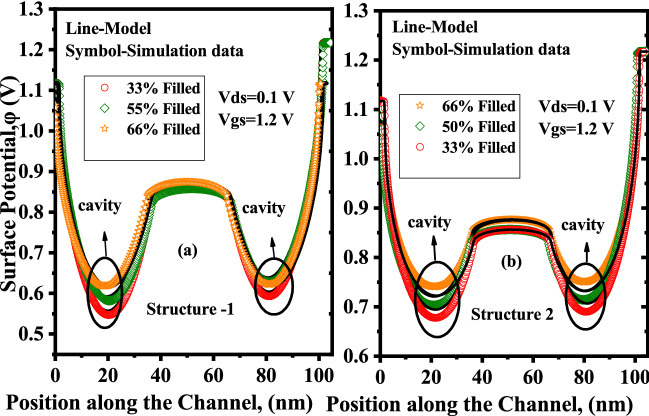
Surface potential of the partially filled cavity (66%,50%,33% filled) ($V_{gs}=0 \; \text{V}$, $V_{ds}=0.1 \; \text{V}$, $\kappa =12$, $N_{f}=0$).

**Figure 14:**
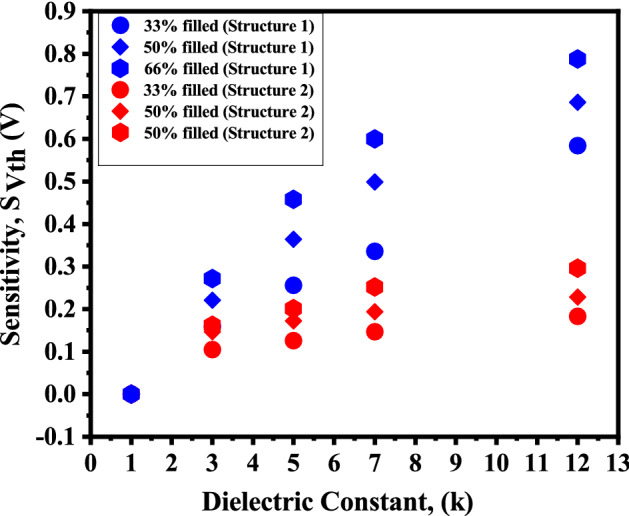
Sensitivity of the proposed DM-DG-TMD-FET-based biosensor when the cavity is partially filled with biomolecules ($V_{gs}=1.2 \; \text{V}$, $V_{ds}=0.1 \; \text{V}$).

Fill-factor of the completely filled cavity is 100%. For the simulation purpose, $L_{1}$ and $L_{2}$ are fixed at 35 nm and $T_{bio}$ is taken to be 6 nm for 66% filled cavity and 4 nm for 50% filled cavity and 2 nm for 33% filled cavity. The effect of partially filled cavities on the surface potential can be understood through (47) and (48). For different percentage filling of profiles (47) is fed to (24) and (25) for Device structure-1 and for Device structure-2 (48) is used and surface potential for partial filled cavity is calculated using (32) and (33). Figure 13 depicts the surface potential for various assumptions of a filled cavity. The figure shows that as $T_{bio}$ is reduced for non-fully filled cavities, the number of biomolecules inside the cavities decreases and the effective gate capacitance decreases, resulting in less gate control. As a result, the source-to-channel barrier increases. Figure 14 shows the sensitivity for different dielectric constants of biomolecules for the fill in factor of 66%, 50% and 33%.

Sensitivity is observed to enhance with increase in fill-factor from 33% to 100% at a specific value of $\kappa$. The maximum sensitivity is shown for a fully filled cavity since the amount of biomolecules conjugation increases as the fill-factor increases. Additionally, it is observed that sensitivity rises as both the fill-factor and the value of $\kappa$ increase.

### Sensitivity analysis due to existence of steric hindrance

Biomolecules are immobilized and hybridized inside the cavity for biosensing operations. Existing hybridized biomolecules block the admission of fresh biomolecules during the process of biomolecule hybridization.

**Figure 15:**
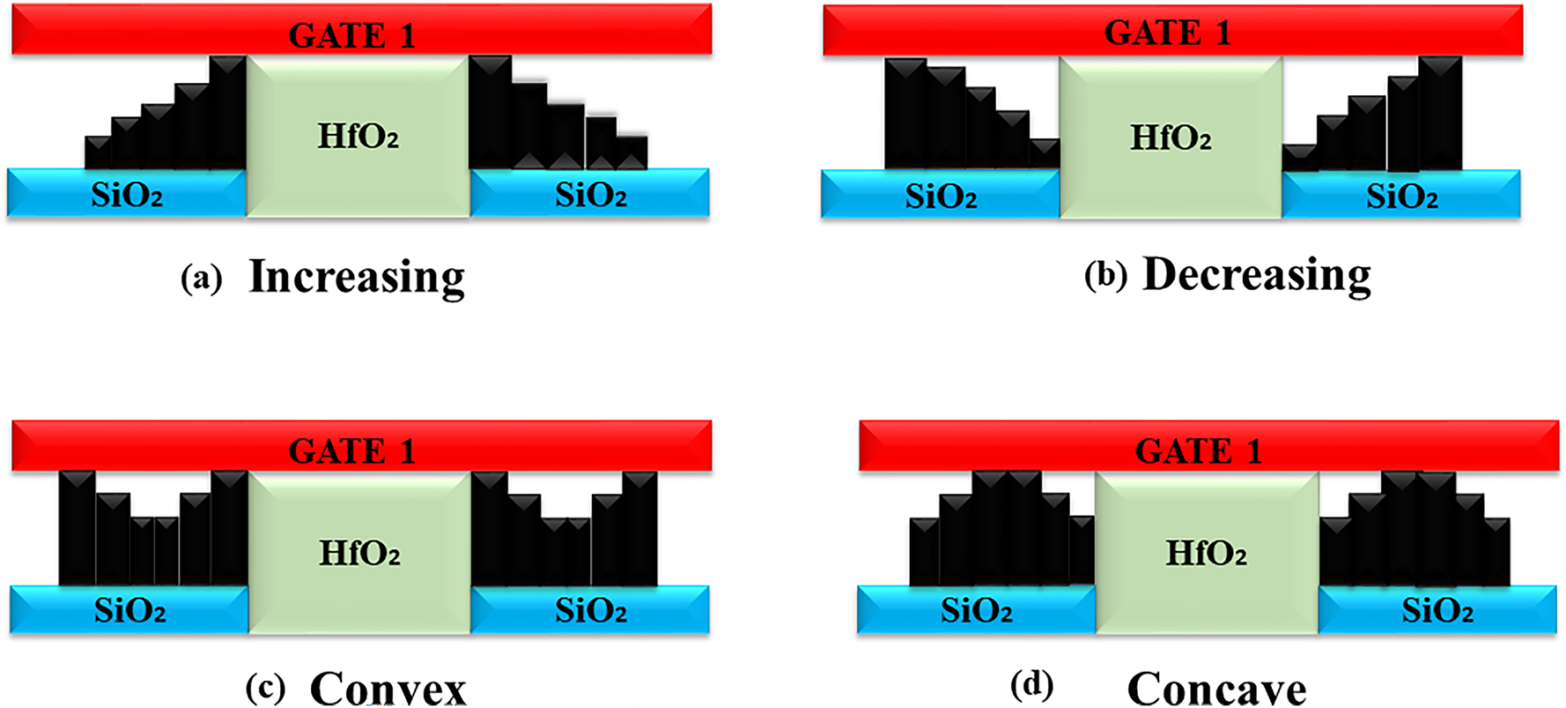
Partially filled cavity profiles (**a**) Decreasing, (**b**) Increasing, (**c**) Convex, and (**d**) Concave.

**Figure 16:**
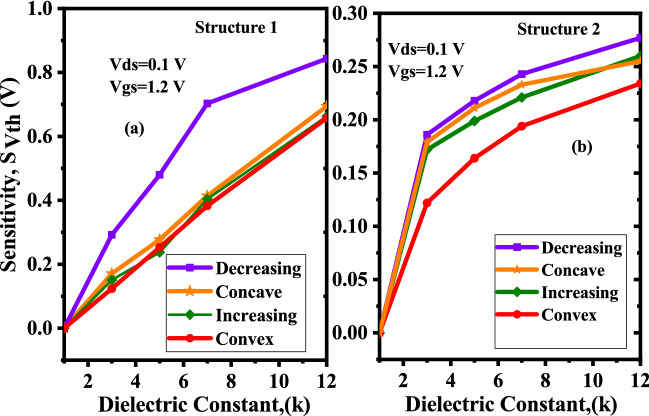
Sensitivity of the proposed DM-DG-TMD-FET-based biosensor for different filling profiles with biomolecules in the cavity ($V_{gs}=1.2 \; \text{V}$, $\kappa =12$, $N_{f=0}$).

This steric hindrance effect may cause biomolecules to hybridize in non-uniform ways along the length of the cavities, according to^7^. Thus, in practical-scenario, completely filled cavities are not conceivable. In this work four different step profiles viz. decreasing, increasing, concave, and convex as shown in Fig. 15a–d to depict the issue of steric hindrance associated with arbitrary and random biomolecular profiles has been considered. A comparison of sensitivities among these profiles has been presented with $\sim$100% filling factor in the cavitites. For decreasing step profile, shown in Fig. 15a, biomolecules $\kappa >1$ will gather nearer to the source/channel junction^7^, and thus coupling between gate and channel enhances. As a consequence, the tunneling rate will speed up and measurable variation on $V_{th}$ happens. Hence, this provide improvement in sensitivity. For the rest of the profiles biomolecules are away from the source to channel interface which reduce the gate to channel coupling and offer poor sensitivities. Decreasing profile of both the structures shows maximum sensitivity. Decreasing profile of Device structure-1 shows sensitivity $S_{Vth}$ of 0.789 V while decreasing profiles of Device structure-2 shows 0.28 V of sensitivity as depicted in Fig. 16a,b.

## Benchmarking

A large number of biosensors based on analytical modelling and simulation are reported in literature. Here, the sensitivity of monolayer DM-DG-TMD-FET-based biosensor is compared with other reported alternative FET-based biosensors. The estimated sensitivities of FET-based biosensors are extracted from literature and compared with this work. Comparative magnitude of sensitivity metrics form different literature is depicted in Table 2. It is observed from Table 2 that the sensitivity of the proposed Device structure-1 with $MoS_2$ as channel material shows highest sensitivity among all the FET-based biosensor structures considered for benchmarking purpose. So, it is envisaged that $MoS_2$-based proposed Device structure-1 can be a potential candidate for biosensing applications with extremely high sensitivity.

**Table 2:** Benchmarking with contemporary works of literature.

Reference	Maximum sensitivity of neutral biomolecules (V)	Maximum sensitivity of charged biomolecules (V)
Ref^32^	0.07	0.085
Ref^33^	0.175	–
Ref^34^	0.09	0.3
Ref^31^	0.22	0.35
Ref^35^	0.318	–
Ref^36^	0.227	0.36
Ref^6^	0.3	–
Ref^23^	0.1	–
Ref^37^	0.2	0.150
Ref^38^	0.150	0.45
Ref^39^	0.11	–
Ref^8^	0.8	–
Our work	0.81	0.5

## Modelling and simulation methods

In^40^, it is shown that 1-D Poisson’s equation can be a rational approximation to explain the electrostatic behavior in 2–D TMD FETs. Surface potential modelling in this work is also based on the solution of the 1-D Poisson’s equation in the channel^40^. Surface potential distribution $\phi (x)$ in the 2–D channel region can be expressed as,

5 $$\begin{aligned} \frac{\delta ^2\Phi _i(x)}{\delta x^2}-P\Phi _i(x)+G=\frac{q}{\varepsilon _{ch}T_{ch}} (N_{A}+n_{2D}(x)) \end{aligned}$$

Where $\varepsilon _{tox}$ and $\varepsilon _{box}$ are top and bottom gate dielectric constants, respectively. Here $T_{tox}$ and $T_{box}$ are top and bottom gate oxide thicknesses respectively, $\varepsilon _{ch}$ is the dielectric constant of 2-D material-based channel. $N_{A}$ is the acceptor type dopant concentration per unit area and $n_{2D}(x)$ is the free inversion carrier concentration. $V'_{Gt}$ and $V'_{Gb}$ are top and bottom gate voltages, respectively defined as,

6 $$\begin{aligned}{} & {} V'_{Gt} = V_{Gt}-V_{Fbt} \end{aligned}$$

7 $$\begin{aligned}{} & {} V'_{Bt} = V_{Gt}-V_{Fbb} \end{aligned}$$

$V_{Fbt}$ and $V_{Fbb}$ are flat band voltages of top and bottom gate, respectively. where,

8 $$\begin{aligned}{} & {} P= \frac{\varepsilon _{tox}}{T_{tox} \varepsilon _{ch} T_{ch}}+\frac{\varepsilon _{box}}{T_{box}\varepsilon _{ch}T_{ch}} \end{aligned}$$

9 $$\begin{aligned}{} & {} G = \frac{\varepsilon _{tox}}{T_{tox} \varepsilon _{ch} T_{ch}}V'_{Gt}+ \frac{\varepsilon _{box}}{T_{box}}\varepsilon _{ch}T_{ch} {V'_{Gb}} \end{aligned}$$

10 $$\begin{aligned}{} & {} n_{2D}(x)= N_{dos} e^{\bigl (\frac{q}{kT}(\Phi (x)-V(x))\bigr )} \end{aligned}$$

Where $N_{dos}$ is the effective density of states of 2-D channel.

Differentiating (5) with respect to *x* and substituting the value of $\frac{q}{\varepsilon _{ch}T_{ch}} (n_{2D}(x))$ from (5) into the result, we obtain,

11 $$\begin{aligned} \frac{\delta ^3\Phi _{i}(x)}{\delta x^3}-P\frac{\delta \Phi _{i}(x)}{\delta x}= \bigg (\frac{\delta ^2\Phi _{i}(x)}{\delta x^2}-K\Phi _{i}(x)+G-\frac{q}{\varepsilon _{ch} T_{ch}} N_{A}\bigg ) \end{aligned}$$

The differential equation in (11) cannot be expressed in a closed form analytical solution. Gradual channel approximation is invoked to simplify (11) and equated as $\frac{\delta V}{\delta x}=0$^41^. (11) can be simplified further by ignoring the variations of higher order $\Phi$ (x) with *x*. As long as the channel is long and drain bias is low, this assumption is valid and it be can expressed as,

12 $$\begin{aligned} \frac{\delta \Phi _{x}}{\delta x}\bigg (\frac{\delta ^2\Phi _{i}(x)}{\delta x^2}-K\Phi _{x}+G-\frac{q}{\varepsilon _{ch}t_{ch}}N_{A}+K \frac{kT}{q}\bigg )=0 \end{aligned}$$

As, lateral electric field is non-zero i.e. $\frac{\delta V}{\delta x}\ne 0$, so, when voltage is applied to the drain, (12) reduces to a linear differential equation as,

13 $$\begin{aligned} \frac{\delta ^2\Phi _i(x)}{\delta x^2}-K\Phi _i(x)=-A \end{aligned}$$

Where,

14 $$\begin{aligned} A= \frac{kT}{q}P+\frac{q}{\varepsilon _{ch} T_{ch}}+G+N_{D} \end{aligned}$$

The closed form solution of the differential equation in (12) can be expressed as,

15 $$\begin{aligned} \Phi (x) = C_{i}e^{\sqrt{x}} + C_{i}e^{\sqrt{x}} + \frac{A_{i}}{K_{i}} \end{aligned}$$

### Modelling of surface potential in overlap region (region II)

In the gate overlap channel region i.e. region II (Fig. 1) Surface potential distribution can be expressed as,

16 $$\begin{aligned} \Phi _{2}(x)= {C_{1}e^{\sqrt{x-{L1}}}} + {C_{2}e^{\sqrt{x-{L1}}}} + {\frac{A_{2}}{P_{2}}} \end{aligned}$$

Following boundary conditions need to be satisfied in this region for the continuity of potential and electric displacement at the interfaces,

17 $$\begin{aligned}{} & {} A_{2}= \frac{kT}{q}K_{2}+\frac{q}{\varepsilon _{ch} t_{ch}}+G_{2}+N_{D} \end{aligned}$$

18 $$\begin{aligned}{} & {} K_{2}= \frac{\varepsilon _{tox}}{T_{tox} \varepsilon _{ch} T_{ch}}+\frac{\varepsilon _{box}}{T_{box}\varepsilon _{ch}T_{ch}} \end{aligned}$$

19 $$\begin{aligned}{} & {} G_{2}= \frac{\varepsilon _{tox}}{T_{tox} \varepsilon _{ch} T_{ch}}V'_{Gt} + \frac{\varepsilon _{box}}{T_{box}\varepsilon _{ch}T_{ch}} {V'_{Gb}} \end{aligned}$$

### Modelling of surface potential in nano-cavity region (region I and region III)

In this subsection, detailed modelling of surface potential in the nano-cavity region (Fig. 1) is discussed. Flatband voltage expressions in the nano-cavity regions *L*1 and *L*3 regions in Fig. 1) can be written as,

20 $$\begin{aligned} V_{fb1}= V_{fb3} =V_{fb2}-\frac{qN_{f}}{C_{eff}} \end{aligned}$$

$V_{fb1}$ and $V_{fb3}$ are the flat band voltages of region I and region III, respectively. Here $C_{fr}$ is the gate electrode fringing capacitance. Fringing-field is modeled using the conformal mapping technique, the mapping functions can be expressed as^42–44^,

21 $$\begin{aligned} (Y-L_{G})+j.nX = M.sinh(v+ju) \end{aligned}$$

**Figure 17:**
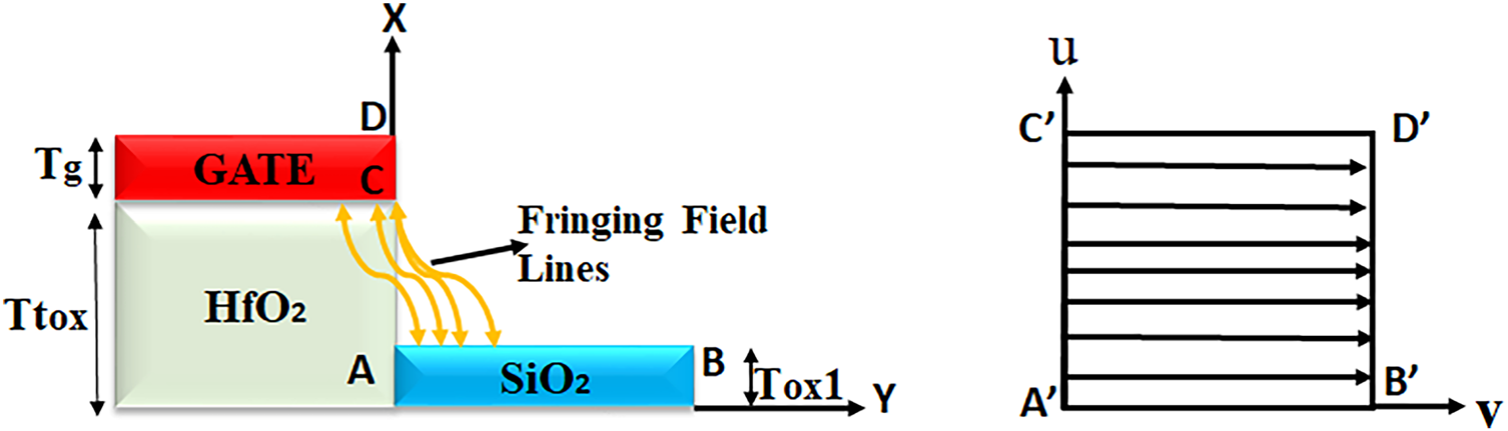
Cross-section of the Device structure 1 (Fig. 1a) showing the fringing field lines and the structural transformation.

The structure after transformation of the device and the orgin of fringing field is shown in Fig. 17. Now using structural transformation technique^42–44^, the fringing capacitance can be expressed as,

22 $$\begin{aligned} C_{fr}= \frac{2\varepsilon _{bio}}{m \pi L_{1,3}} sinh\bigg (acosh\bigg (\frac{(T_{ox}-T_{ox1})+T_{g}}{T_{ox}-T_{ox1}}\bigg ) \end{aligned}$$

### Modelling of surface potential in nano-cavity region (region I and region III) for Device structure-1

For the proposed DM-DG-TMD-FET structure-1 mentioned in (Fig. 1a), when the fringing fields are dominant, the effective capacitance is determined primarily by the fringing capacitance.

23 $$\begin{aligned} C_{eff}= \frac{C_{ox1}C_{fr}}{C_{ox1}+C_{fr}} \end{aligned}$$

So, for the region I ($L_{1}$) of the Device structure-1 shown in (Fig. 1a), the closed form expression of surface potential can be written as

24 $$\begin{aligned} \Phi _{1}(x)={C_{3}e^{\sqrt{x}}} + {C_{4}e^{\sqrt{x}}}+ \frac{A_{1}}{P_{1}} \end{aligned}$$

For region III ($L_{3}$), the closed form expression of surface potential can be written as,

25 $$\begin{aligned} \Phi _{3}(x)= {C_{5}e^{\sqrt{x-L_{1}-L_{2}}}} + {C_{6}e^{\sqrt{x-L_{1}-L_{2}}}}+ {\frac{A_{3}}{P_{3}}} \end{aligned}$$

Where,

26 $$\begin{aligned}{} & {} A_{1}= \frac{kT}{q}K_{1}+\frac{q}{\varepsilon _{ch} T_{ch}}+G_{1}+N_{D} \end{aligned}$$

27 $$\begin{aligned}{} & {} P_{1}= \frac{C_{fr}}{ \varepsilon _{ch} T_{ch}}+\frac{C_{fr}}{\varepsilon _{ch}T_{ch}} \end{aligned}$$

28 $$\begin{aligned}{} & {} G_{1}= \frac{C_{fr}}{ \varepsilon _{ch} T_{ch}}V'_{Gt} + \frac{C_{fr}}{\varepsilon _{ch}T_{ch}} {V'_{Gb}} \end{aligned}$$

29 $$\begin{aligned}{} & {} A_{3}= \frac{kT}{q}K_{3}+\frac{q}{\varepsilon _{ch} T_{ch}}+G_{3}+N_{D} \end{aligned}$$

30 $$\begin{aligned}{} & {} P_{3}= \frac{C_{fr}}{\varepsilon _{ch} T_{ch}}+\frac{C_{fr}}{\varepsilon _{ch}T_{ch}} \end{aligned}$$

31 $$\begin{aligned}{} & {} G_{3}= \frac{C_{fr}}{ \varepsilon _{ch} T_{ch}}V'_{Gt} + \frac{C_{eff}}{\varepsilon _{ch}T_{ch}} {V'_{Gb}} \end{aligned}$$

### Modelling of surface potential in nano-cavity region (region I and region III) for Device structure-2

For region I ($L_{1}$) of the Device structure-2 in (Fig. 1b), the closed form expression of surface potential can be written as,

32 $$\begin{aligned} \Phi _{1}(x)={C_{3}e^{\sqrt{x}}} + {C_{4}e^{\sqrt{x}}}+ \frac{A_{1}}{P_{1}} \end{aligned}$$

For region III ($L_{3}$), the closed form expression of surface potential can be written as,

33 $$\begin{aligned} \Phi _{3}(x)= {C_{5}e^{\sqrt{x-L_{1}-L_{2}}}} + {C_{6}e^{\sqrt{x-L_{1}-L_{2}}}}+ {\frac{A_{3}}{P_{3}}} \end{aligned}$$

Where,

34 $$\begin{aligned}{} & {} A_{1}= \frac{kT}{q}K_{1}+\frac{q}{\varepsilon _{ch} t_{ch}}+G_{1}+N_{D} \end{aligned}$$

35 $$\begin{aligned}{} & {} P_{1}= \frac{C_{eff}}{ \varepsilon _{ch} T_{ch}}+\frac{C_{eff}}{\varepsilon _{ch}T_{ch}} \end{aligned}$$

36 $$\begin{aligned}{} & {} G_{1}= \frac{C_{eff}}{ \varepsilon _{ch} T_{ch}}V'_{Gt} + \frac{C_{eff}}{\varepsilon _{ch}T_{ch}} {V'_{Gb}} \end{aligned}$$

37 $$\begin{aligned}{} & {} A_{3}= \frac{kT}{q}K_{3}+\frac{q}{\varepsilon _{ch} T_{ch}}+G_{3}+N_{D} \end{aligned}$$

38 $$\begin{aligned}{} & {} P_{3}= \frac{C_{eff}}{\varepsilon _{ch} T_{ch}}+\frac{C_{eff}}{\varepsilon _{ch}T_{ch}} \end{aligned}$$

39 $$\begin{aligned}{} & {} G_{3}= \frac{C_{eff}}{ \varepsilon _{ch} T_{ch}}V'_{Gt} + \frac{C_{eff}}{\varepsilon _{ch}T_{ch}} {V'_{Gb}} \end{aligned}$$

For the proposed DM-DG-TMD-FET structure-2 mentioned in (Fig. 1b), when the fringing fields are less dominant, the effective capacitance is determined primarily by the gate capacitance.

40 $$\begin{aligned} C_{eff}= \frac{C_{ox1}C_{gap}}{C_{ox1}+C_{gap}} \end{aligned}$$

Here, $C_{gap}$ is the capacitance of cavity region and it can be expressed as,($\frac{\varepsilon _{bio}}{T_{cav}}$). For finding out the constant coefficients, $C_{1}$, $C_{2}$, $C_{3}$, $C_{4}$, $C_{5}$ and, $C_{6}$, following boundary conditions are enforced.

41 $$\begin{aligned}{} & {} \Phi _{1}(x=0)=V_{s}+V_{bi}+\frac{KT}{q} \ln \bigg (\frac{N_{sd}}{N_{dos}}\bigg ) \end{aligned}$$

42 $$\begin{aligned}{} & {} \Phi _{1}(x=L_{1})= \Phi _{2}(x=L_{1}) \end{aligned}$$

43 $$\begin{aligned}{} & {} \frac{\delta \Phi _{1}(x)}{\delta x}= \frac{\delta \Phi _{2}(x)}{\delta x} at (x=L_{1}) \end{aligned}$$

44 $$\begin{aligned}{} & {} \Phi _{2}(x=L_{2})= \Phi _{3}(x=L_{2}) \end{aligned}$$

45 $$\begin{aligned}{} & {} \frac{\delta \Phi _{2}(x)}{\delta x}= \frac{\delta \Phi _{3}(x)}{\delta x} at (x=L_{2}) \end{aligned}$$

46 $$\begin{aligned}{} & {} \Phi _{3}(x=L_{3})= V_{d}+V_{bi}+\frac{KT}{q} \ln \bigg ( \frac{N_{sd}}{N_{dos}}\bigg ) \end{aligned}$$

Solving (42), (43), (44) and, (45), the constant coefficients can be evaluated and then substituting the values of constants in (24), (25), (32) and (33) surface potential can be evaluated.

### Surface potential modelling for partially filled cavity

In this section, surface potential is modeled for the mentioned device structures in (Fig. 1) with partially filled nano-cavity which is filled upto a certain height, $T_{bio}$. For Device structure-1 (Fig. 1a) $T_{cav}$=$T_{bio}$ considered as 10 nm and for Device structure-2 (Fig. 1b) as 9 nm (this is for fully filled case). For partially field case, $T_{bio}$ can be treated as a parameter. Gate electrode fringing capacitance when the nano-cavity of region I and region III is partially filled upto a certain height ($T_{bio}$) can be expressed as,

47 $$\begin{aligned} C_{fr}= \frac{2\varepsilon _{bio}}{m\pi L_{1,3}} sinh\bigg (acosh\bigg (\frac{(EOT-T_{ox}-T_{ox1})+T_{g}}{EOT-T_{ox}-T_{ox1}}\bigg )\bigg ) \end{aligned}$$

Where

48 $$\begin{aligned} EOT= T_{bio}+ \frac{\varepsilon _{bio}}{\varepsilon _{air}} (T_{cav}-T_{bio}) \end{aligned}$$

where $T_{bio}$ is the height of biomolecules occupancy and $\varepsilon _{bio}$ is the dielectric constant of biomolecules. By substituting (47) in (24) and in (25) surface potential for partially cavity case can be obtained. For the proposed Device structure-2, effect of (48) will get added to (40) and from (32) and (33), surface potential for partially filled cavity case can be obtained.

### Drain current modelling

Model of drain current in subthreshold region is calculated by utilizing expressions of surface potential obtained in previous section^45^ and can be expressed as,

49 $$\begin{aligned} I_{dsub} = \frac{\mu WkT \frac{N^2_{i}}{N_{dos}} \Biggl (1- e^{\frac{-qV_{ds}}{kT}}\Biggr )}{\sum _{i=1}^{3} \int _{0}^{L_{i}} e^{\frac{q\Phi _{i}(x)}{kT}}dx} \end{aligned}$$

For linear regime, three transistor modelling approach has been employed to calculate the drain current^46^. Both the subthreshold current and linear regime current have been equated at transition region for the continuity of the current. The carrier transport is governed by the drift-diffusion equation described as,

50 $$\begin{aligned} I_{ds}= qWn_{2D}(x)\mu (x)\frac{\delta V(x)}{\delta x} \end{aligned}$$

Where $\mu (x)$ is carrier mobility in the channel, and *W* is device width. Here an extraction of *V*(*x*) in terms of *x* to calculate the drain current is required. We assume a linear profile of *V*(*x*) and simplified expression of potential can be written as

51 $$\begin{aligned} V(x)=M(x)+C \end{aligned}$$

The constants *M* and *C* can be evaluated as mentioned in^23^. To incorporate the effect of gate bias, an empirical fitting function $F(V_{G})$ can be considered with *C*. So, the final form of *C* can be expressed as,

52 $$\begin{aligned} C= V_{s}+V_{bi}+F(V_{G}) \end{aligned}$$

Now integrating (50) w.r.t to *x*, current in each region can be evaluated. The drain current in region I,II,III (Fig. 1) is the drain current of the DM-DG-TMD-FET with channel length, $L_{1}$, $L_{2}$, and $L_{3}$ respectively, and it can be expressed as,

53 $$\begin{aligned} I_{dlinear,i}= \frac{q \mu _{n}N_{dos}}{L_{i}}\int _{0}^{L_{i}} exp\bigg ( \frac{q}{kT}(\Phi _{i}(x)+\frac{A}{K}-Mx-C)\bigg )dx \end{aligned}$$

Where, $$i= 1,2,3$$.

### Simulation methodology

The developed model of the TMD FET-based biosensor has been validated with the 2-dimensional numeric simulator SILVACO TCAD^25^. Atomistic simulators such as Nano TCAD ViDES is compatible for atomistics simulations of TMD devices but they suffer from computational complexity and it has its own limitations. Moreover, many recent work based 2-D materials FET have been using SILVACO TCAD for simulating FETs^18,47^. In SILVACO TCAD, while defining 2-D materials, the electrical properties of materials like permittivity, densitiy of states, electron and hole effective masses, affinity, electron and hole mobilities need to be defined. For simulation of the proposed device we have adopted the fermi-dirac carrier distribution, the electric field-dependent mobility (FLDMOB) model concentration dependent mobilty (CONMOB) model. Shockley- Read-Hall recombination model is also included for the recombination mechanism. As the source and drain regions are highly doped regions, a significant amount of band bending is there in these regions due to high doping, to include this in calculations, Band-gap narrowing model(BGN) has also been considered. The effect of neutral biomolecules is modeled by introducing a dielectric materials having dielectric constant ($\kappa$) varied between 1 to 12, which lies in the range of dielectric constant of different biomolecules (e.g., Protein ($\kappa$ = 2.50), Biotin ($\kappa$  = 2.63), APTES ($\kappa$  = 3.57), Protein ($\kappa$  = 6) and Streptavidin ($\kappa$ = 2.1)^5,48^). Negative and positive charge density ($N_{f} = -1\times 10^{-12}$ $\text{C}/\text{m}^2$ to $+1\times 10^{11}$ $\text{C}/\text{m}^2$) at the $MoS_{2}/SiO_{2}$ interface is considered to model the impact of charged biomolecules. Material parameters of the TMD materials have been extracted from^47^.

## Conclusion

In this work, for monolayer $MoS_{2}$-based dual-gate FET-based biosensor, an analytical model has been developed including the fringing-field effect. The model is developed in such a way that it can be utilized to design DM-DG-TMD FET-based biosensor based on the principle of modulation of dielectric constants. The proposed model can be applied to any TMD material to measure its impact on sensitivity. The proposed biosensor shows $\sim 100\%$ higher sensitivity in Device structure-1 compared to Device structure-2. Channel surface potential and transfer characteristics obtained from the model show a quantifiable variation in sensor output with the variation of dielectric constant in the nano-cavity region. The effect of partially filled cavities has been also modeled and investigated in this work. Steric hindrance issue has also been studied in this work. The results demonstrate that the completely filled cavity shows $\sim 92\%$ higher sensitivity than the partially filled cavities (i.e. 33% filled cavity). Optimization of device dimensions has also been done to enhance the sensitivity of the proposed biosensors. Finally, benchmarking of the proposed biosensor is presented with contemporary, state of the art biosensors and it is highlighted that $MoS_{2}$ FET-based biosensor emerges with a superior sensitivity $S_{Vth}$ = 0.81 V for $\kappa =12$. Finally, it is concluded that our proposed DM-DG-TMD FET-based Biosensor structure-1 model is an extremely promising candidate for the biosensing applications due to its enhanced sensitivity and label-free detection trait which could be immensely useful for detection of lower concentration of biomolecules.

## Data Availability

Data can be available upon reasonable request to the corresponding author.
